# Cost Analysis of a Digital Multimodal Cancer Prehabilitation

**DOI:** 10.3390/curroncol29120729

**Published:** 2022-11-29

**Authors:** Evdoxia Gkaintatzi, Charoula Konstantia Nikolaou, Tarannum Rampal, Roberto Laza-Cagigas, Nazanin Zand, Paul McCrone

**Affiliations:** 1 Institute for Lifecourse Development, University of Greenwich, London SE10 9LS, UK; 2 Natural Resources Institute, University of Greenwich, London SE10 9LS, UK; 3 Kent and Medway Prehab, Phase-B CIC, London E1 6RA, UK

**Keywords:** prehabilitation, cancer, healthcare costs, quality-adjusted life years

## Abstract

Introduction: There is growing evidence that prehabilitation programmes effectively improve the physical and psychological conditions of cancer patients awaiting treatment. During the pandemic, people with cancer were classed as vulnerable. To reduce risk to this population Kent and Medway Prehabilitation service transformed into a TeleHealth format. The aim of this study is to assess the impact on health-related quality of life (HRQoL) and the costs of a digital multimodal prehabilitation programme. Methods: HRQoL was measured with the EQ-5D and quality-adjusted life years (QALYs) were calculated. Costs of the prehabilitation service and inpatient care were calculated. Comparisons were made between different levels of prehabilitation received. Results: A sample of 192 individuals was included in the study Mean HRQoL improved from 69.53 at baseline to 85.71 post-rehabilitation, a 23% increase. For each additional week of prehabilitation care in cancer patients, the model predicts that the total QALYS increase by 0.02, when baseline utility is held constant. Conclusions: Prehabilitation is associated with improved HRQoL and QALYs. Our model of a multimodal digital prehabilitation program can be beneficial for patients and reduce costs for healthcare facilities even when the patients attend only a few sessions.

## 1. Introduction

The past few decades have observed significant advancements in cancer treatment. The treatment developments mean that many more cancer patients have improved survival rates and chances for remission and cure [1,2]. The period between a patient being diagnosed and starting treatment has been identified as a critical period for the success of any of the treatments [3,4]. Cancer patients with optimal physical and psychological function ability present improved results from treatments and therefore have improved survival rates. Accordingly, cancer prehabilitation, a term coined by Silver & Baima in 2013 [5], uses the period before the initiation of the treatment to correct any functional and psychological decline that occurred before the diagnosis and prevent any further functional and psychological decline and its subsequent consequences.

During the COVID-19 pandemic, a lot of health care services were forced to move online at a short notice including cancer prehabilitation services. Digital health programmes have lower costs compared to face-to-face programmes and can accommodate a more significant number of patients simultaneously. Additionally, there is potential for the adoption and delivery of the model across different geographical locations. Cancer prehabilitation programmes can be multi-modal or unimodal. The multi-modal programmes usually include physical activity, nutrition, and psychological support component, while the unimodal programmes more often offer either physical activity or a nutrition programme. A growing body of evidence shows that cancer prehabilitation programmes can improve exercise capacity, reduce hospital stay and present a trend for better quality of life [6,7,8,9]

Furthermore, cancer prehabilitation can support cancer patients to carry on with their pre-diagnosis activities post-treatment [8]. While we have evidence on the effectiveness of prehabilitation programmes, we only have few studies looking into the cost of these programmes. A recent study showed that the programme costed EUR 389 per patient and showed a protective effect for 30-day hospital readmissions [10]. Prehabilitation programme in the form of preoperative physiotherapy was cost-effective with an incremental net benefit to participating hospitals of AUD 4958 [11]. Another study showed that prehabilitation programme generated a mean cost saving per patient of EUR 3092 [12].

The study aims to explore the impact of a digital prehabilitation programme for cancer patients in the South of England, previously validated for its effectiveness [13] on health-related quality of life (HRQoL), variability of dose, costs, and inpatient costs.

## 2. Methods

### 2.1. Digital Intervention

The intervention was affected by the COVID-19 pandemic, which altered the delivery of prehabilitation services from centre-based and face-to-face to remote, based on digital technology and telecommunication methods, in order to shield the high-risk and medically vulnerable patients. Thus, the intervention can be described as a multimodal digital programme offering guidance on physical activity, nutrition, lifestyle and psychology. The eligible patients were either self-referred or referred by a healthcare professional to a regional prehabilitation unit that provides home-based services. Referrals are completed using the service’s webpage. The specialties of cancers that the recruited patients suffered from were colorectal, liver, urologic (bladder, kidney, and prostate), lung, breast, head and neck and brain cancer. The cancer treatments provided were either a single therapy or a combination of the methods: chemotherapy, surgery, radiotherapy, hormone therapy, immunotherapy, or active monitoring, accompanied by prehabilitation services. In surgical oncology pathway, patients were offered prehabilitation services until the point of surgery while for patients receiving either chemotherapy or radiotherapy, the services were offered before and during the course of the treatment. Specialists and counsellors of the programme completed remote consultations with patients, assessing their psychological and physical needs. The Quality of Life (QoL) was obtained through the Digital questionnaire that was sent via the programme’s app or email to the patients. Craetus platform was used to create a supervised, targeted plan based on patient’s functional capacity and clinical need. The number of sessions conducted was different for each patient and based on personal needs. More details on the patients’ recruitment and prehabilitation activities can be found in the paper published on the effectiveness of the programme [8].

### 2.2. Measures 

The data were collected on the basis of five time-points (T1–T5) during each patient’s treatment pathway described in Table 1. The time points differ according to each patient’s treatment. Some patients only have one measurement of a specific scale while others have up to five. Data on demographics (e.g., age, gender, ethnic group), participant’s health status, treatment pathway and lifestyle choices (e.g., nutrition, smoking) were obtained from healthcare records, EQ-5D scores [9] from patient’s questionnaires and costs from both Kent and Medway team and the literature.

The EQ-5D questionnaire rated patients’ health levels in the following 5 dimensions: mobility, self-care, usual activities, pain/discomfort, and anxiety/depression at each different time points (T1–T5) during their treatment pathway, as mentioned above. The tool scores each dimension with an integer value between 1, representing the absence of problem in that area, to 5, related to extreme problem experience in the area. The answers given by the patients for the five domains are then used to generate a summary score, which indicated the overall state utility. The cohort was also asked to self-evaluate their general health and fatigue levels on the EQ-5D visual analogue scale of 1 to 100.

Quality Adjusted Life Years (QALYs) were used as the measurement to encapsulate the impact of the prehabilitation services both on the length of life and on the health-related quality of life (QoL). The use of QALYs is required by the National Institute for health and Clinical Excellence (NICE) in the United Kingdom to judge the cost effectiveness of new technologies. For QALYs generation, utility scores are needed. Utilities are preference weights and hence, different utility scores define different health states of the patients. The QoL is expressed as a utility value on a scale of 0, which indicates the worst health state (death) to 1, which indicates full health. Ultimately, the aim is to estimate the cost per QALY, which is defined as the cost of each additional life year by an induced intervention not only in terms of additional lifetime but also in terms of life quality, expressed as reduced pain or normal daily function. Currently, for its standard technology appraisals, NICE uses a threshold of GBP 20,000 to GBP 30,000 per QALY, meaning that innovative interventions that come below the threshold are usually approved.

### 2.3. Analysis

Data were entered and analysed using Microsoft Excel, the Statistical Package for the Social Sciences (SPSS), and Stata. The EQ-5D scores were converted to utility weights using an algorithm within Stata [14].

Quality-adjusted life years (QALYs) were calculated for those with utility scores at each time point using the area under the curve method, which assumes a linear change between adjacent scores. Changes in average self-rated health and the EQ-5D utility scores were compared over time. Four bands of prehabilitation service duration of use were formed (band 1: 0–2.99 weeks of prehab, band 2: 3–4.99 weeks of prehab, band 3: 5–10.5 weeks of prehab and band 4: over 10.5 weeks of prehab). The goal was to facilitate the comparison of utility scores and QALYs between these bands and reveal a potential health improvement.

The cost of Prehab services and inpatient care were calculated, in combination with the duration of prehabilitation use and the length-of-stay variables, respectively, provided in the dataset. The costs related to Prehab services were obtained from the healthcare provider while the cost of inpatient care per day from the literature [15]. The inpatient care refers to the hospital stays regarding the patient’s cancer surgery. The suggested value was reported to 2008 prices in euros, and it was uplifted and exchanged to a 2022 value of GBP 555 per hospital day. The Prehab, inpatient and total costs were also assessed in terms of prehabilitation service duration and the cancer speciality of each participant.

Finally, a multiple regression analysis was performed to analyse the relationship between QALYs and two independent variables: baseline utility score and duration of prehabilitation (in weeks).

## 3. Results

A sample of 192 individuals who were referred to the Kent and Medway Prehab service, as part of their cancer therapy, were included in the study. Demographic information for the sample is presented in Table 2. Male patients accounted for two-thirds of the recruited population and their mean age was 67 years. On the contrary, the mean age of the female population included was 62 years. Colorectal and urological types of cancer were the main diagnosis for 82% of the sample size. Furthermore, approximately 70% of the patients underwent a surgery, as the main cancer treatment, following those who received combination therapy (13%) and monotherapy (11.5%). The adherence rate to the programme measured both through the app and discussions with the physiologist at patients appointment was 85%.

**Figure 1:**
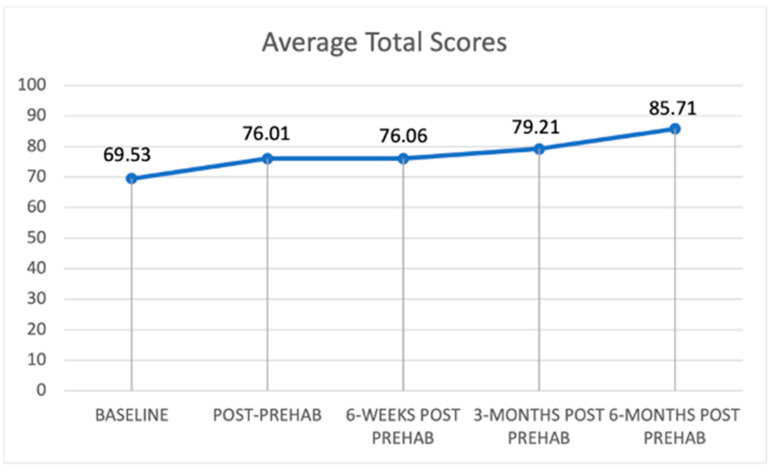
Trajectory of self-reported health-related quality of life.

**Figure 2:**
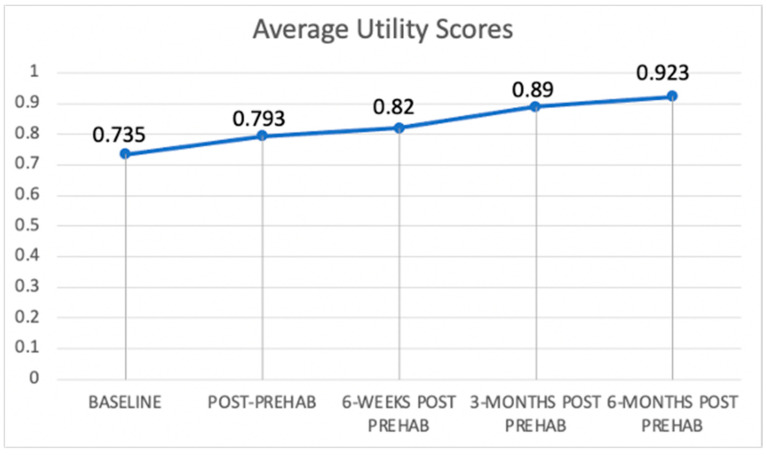
Trajectory of EQ-5D utility scores.

Figure 1 and Figure 2 present trajectories of the self-rated health-related quality of life and EQ-5D utility score, respectively, which are both increasing for patients over the selected time points (Figure 1 and Figure 2). This indicates general health improvement, as they continue to receive a combined therapy option of cancer treatment and prehabilitation services. Figure 1 shows that the increase is around 16 points (or 23%) over time. Figure 2 shows an increase in average utility values of 0.188 (or 26%).

Average utility scores and QALYs of participants were assessed in relation to the duration of their prehabilitation service use. Table 3 shows that the average QALYs were higher (0.73) for higher levels of prehabilitation received in comparison to the lower bands (0.48, 0.5 and 0.57, respectively). This suggests a clear effect of prehabilitation. Similarly, Table 3 reveals the average utility scores by prehabilitation use band. The scores at 6 months (post-intervention) were noticeably higher (0.99) for those receiving the highest amount of prehabilitation care (over 10.5 weeks of prehabilitation use).

Regarding the regression analysis conducted (Table 4), both independent variables were statistically significant predictors of QALYs. For each additional week of prehabilitation care in cancer patients, the model predicts that the total QALYS increase by 0.02, when baseline utility is held constant. A unit increase in baseline utility score will lead to a QALYs increase of 0.26.

Finally, the mean costs associated with cancer prehabilitation services and inpatient care were estimated and presented in Table 4, accompanied by their sum. Inpatient care costs are approximately 10 times higher than prehabilitation-related ones.

Even though colorectal cancer presents the lowest prehabilitation costs, it is associated with high inpatient care costs unlike patients diagnosed with urological cancers (Table 5). Furthermore, as the duration of prehabilitation use by the patients increase, the associated costs are also increasing (Table 6). Table 6 shows that the inpatient costs and prehabilitation service duration are disproportionate, leading to the general conclusion that the longer use of prehabilitation is linked with lower inpatient expenses for the NHS. However, the higher inpatient costs are related to cancer patients that have utilized the Prehab services from five to ten and a half weeks.

## 4. Discussion

To the best of our knowledge, this is the first study exploring the variability of dose effect of a cancer prehabilitation multimodal digital programme. It also includes information on the patients’ health-related quality of life, the prehabilitation programme’s cost, and inpatient care costs. Patients participating in the prehabilitation cancer service showed an improvement in self-reported quality of life, an increase in the EQ-5D utility scores, and an increase in QALYs for more extended periods of prehabilitation.

Our study included patients with different forms of cancer. A recent review of studies on cancer prehabilitation found an improvement in the reported quality of life of patients with prostate cancer with no progress in functional ability or distress and an improvement in the quality of life of breast cancer patients [9]. A Cochrane review looking at prehabilitation programmes for colorectal patients found an improved functional capacity and fewer complications, but studies included in the review did not report outcomes in quality of life and overall were of low quality, so the certainty of the evidence was also low [16].

The duration of prehab programmes differs a lot among studies and countries. In a review of prehabilitation programmes focused only on physical activity, the time of them ranged from 7 to 52 days, with a median duration of 21 days [17]. Multimodal prehabilitation programmes last between 4–8 weeks, with most programmes recommending four weeks. In a recent randomised controlled trial with patients diagnosed with lung cancer, a two-week prehabilitation programme was trialed to increase compliance and significantly improve the six-minute walking distance test in the intervention group [18]. In the UK, guidelines state that patients should not have to wait more than 31 days from the diagnosis to the initiation of treatment. Our study showed that even a small number of prehab sessions improved quality of life, ED-5D utility scores and QALYs. Patients having non-surgical oncology management continued with sessions during their treatment. This way, the patients were confident that their treatment would not be delayed until a set number of prehab sessions were completed. Additionally, at this time that there are long waiting lists for treatment and often the 31-day target is not achieved, prehab services are a great way for patients to invest their time through improving health related quality of life and fitness.

The average cost of the prehab service was estimated to be GBP 460, significantly lower than the ten times higher inpatient costs. The cost is also significantly lower compared to other prehabilitation services. A large study with 4415 women with ovarian cancer estimated that the cost of care with prehabilitation per patient was USD 84,053 while the cost of usual care was USD 91,713. Sensitivity analysis showed that prehabilitation was more cost-effective at USD 9418/patient [19].

This study has several strengths that deserve to be mentioned here. To the best of our knowledge, this is the first study looking into the variability in dose of cancer prehabilatation for patients. The mode of delivery was digital, removing some barriers to service access, even for patients who may have been feeling unwell or did not have the means to travel to the service. COVID-19 outbreak at the time of the study might have increased the number of participants that did not have any other alternative due to services being paused. The flexibility given to patients in terms of the location of the programme and the number of sessions increases compliance with the programme. The availability of a remote prehabilitation programme was not a barrier for patients in our case, but it resulted in reduced transport costs and improved time efficiency.

Potential weaknesses of our study include the lack of a control group and the relative non-homogenous group of participants with different types of cancer and treatment. However, the enrolment of all patients in the study reduced the chance of selection bias. As this was a preliminary study, there was no formal sample size calculation, so there might be a lack of power to detect slight differences. COVID-19 outbreak may have had a negative impact on results as the lockdown had both psychological and physical effects on the population and therefore might have diluted our results. The lack of a control group means that we do not assess cost-effectiveness and the findings are largely descriptive. Providing such cost information aims to highlight that prehabilitation services reduce the spending on inpatient care, which is highly informative for future research. Another weakness was that we did not differentiate between severity of cancer in our population as this was a service offered to anyone as long as they had a cancer diagnosis.

Our findings support that a digital prehabilitation programme could be an effective mode of intervention for some patients. Future more extensive and adequately powered studies with a control group will help further evaluate digital prehabilitation programmes’ effects on treatment, physical and psychological outcomes, quality of life, and QALYs. NHS’s long-term strategy [20] is to move chronic conditions patients and about a third of outpatients’ appointments in a digital environment. Moreover, the current waiting list and staff shortages mean there needs to be a radical reform of what secondary care is for we have presented a feasible, effective, and cost-effective model for tackling cancer waiting lists.

## 5. Conclusions

Our findings suggest that our model of a multimodal digital prehabilitation program comprising physical activity, nutrition and psychological support can be beneficial for patients and reduce costs for healthcare facilities even when the patients attend only a few sessions.

## Figures and Tables

**Table 1:** Time points of data collection.

Treatment	T1	T2	T3	T4	T5
Surgery	Baseline	1–3 days before surgery	6 weeks post-surgery	3 months post-surgery	6 months post-surgery
Radio-/Chemotherapy	Baseline	7–10 days after the last treatment session	6 weeks after T2	3 months after T2	6 months after T2

**Table 2:** Characteristics of the participants.

	Value (%)	Sample Size
Specialty of diagnosed cancer		192
Colorectal	108 (56.3)	
Urologic	50 (26.0)	
Breast	15 (7.8)	
Lung	3.6 (2)	
Brain	4 (2.1)	
Head & Neck	5 (2.6)	
Pancreatic	1 (0.5)	
Prostate	2 (1)	
Gender		192
Female	74 (38.5)	
Male	118 (61.5)	
Mean Age (Years)		
Female	61.77	74
Male	66.92	118
Treatment received		192
Surgery	134 (69.8)	
Hormone therapy + Radiotherapy	1 (0.5)	
Palliative care	1 (0.5)	
Surgery + Radiotherapy	2 (1)	
Chemotherapy	10 (5.2)	
Chemotherapy + Surgery	7 (3.6)	
Hormone therapy	4 (2.1)	
Active monitoring	1 (0.5)	
Chemoradiotherapy + 2 surgeries	1 (0.5)	
Radiotherapy	8 (4.2)	
Chemoradiotherapy	10 (5.2)	
Chemoradiotherapy + surgery	3 (1.6)	
Radiotherapy + immunotherapy	1 (0.5)	
Treatment received (%)		
Surgery	69.8	
Single therapy	11.5	
Combination therapy	12.9	
Palliative care	0.5	
Active Monitoring	0.5	
Mean Prehabilitation duration (weeks)	8.47	177
Mean Length of hospital stay (days)	8.14	97
Referred by a HC professional	168	192
Self-referred	24	
Adherence rate	85%	192
Acceptance rate	76 %	192

**Table 3:** Average QALYs per cancer type group or prehabilitation duration band.

Types of cancer	Average QALYs
Colorectal	0.537
Urologic	0.666
Breast	0.492
Lung	0.446
Brain	- *
Head & Neck	0.273
Pancreatic	- *
Prostate	- *
**Prehab Duration Band**	
0–2.99 weeks of prehabilitation use (*N* = 11)	0.48
3–4.99 weeks of prehabilitation use (*N* = 11)	0.5
5–10.5 weeks of prehabilitation use (*N* = 9)	0.57
Over 10.5 weeks of prehabilitation use (*N* = 13)	0.73

*: no data available.

**Table 4:** Average total QoL in each prehab duration of use band (in weeks).

	0–2.99 Weeks of Prehabilitation Use	3–4.99 Weeks of Prehabilitation Use	5–10.5 Weeks of Prehabilitation Use	Over 10.5 Weeks of Prehabilitation Use
Average Utility Score: Post rehab	0.83	0.78	0.76	0.79
Average Utility Score: 6 weeks post rehab	0.83	0.82	0.85	0.78
Average Utility Score: 3 months post rehab	0.88	0.9	0.86	0.88
Average Utility Score: 6 months post rehab	0.89	0.86	0.99	0.99

**Table 5:** Multivariate regression analysis with the dependent variable to be total QALYs.

Regression Weights	Unstandardized β-Coefficient	R^2^	F	*p*-Value
Utility score-baseline *	0.26	0.8	97.6	<0.001
Prehab duration (in weeks) *	0.02			<0.001

* The baseline utility score and the duration of prehabilitation (in weeks) explain 80% of the variability of Total QALYs.

**Table 6:** Mean prehabilitation, inpatient and total cost of participant groups, according to their cancer diagnosis and total costs in each Prehab use duration band (in weeks).

	Prehabilitation Costs (GBP)	Inpatient Costs (GBP)	Total Costs (GBP)
Mean costs (GBP)	460	4520	4856
Colorectal Cancer patients	314	5249	5563
Urological Cancer Patients	750	2337	3087
Other type of cancers patients	471	3330	3801
0–2.99 weeks of prehabilitation	103	4500	4603
3–4.99 of prehabilitation use	212	4440	4652
5–10.5 weeks of prehabilitation use	397	5170	5567
Over 10.5 weeks of prehabilitation use	1153	3950	5103

## Data Availability

The data presented in this study are available on request from the corresponding author. Data is not publicly available due to privacy concerns.
